# Characterization of SHH, SOX3, WNT3A and WNT9B Proteins in Human Non-Syndromic Cleft Lip and Palate Tissue

**DOI:** 10.3390/dj11060151

**Published:** 2023-06-09

**Authors:** Mārtiņš Vaivads, Ilze Akota, Māra Pilmane

**Affiliations:** 1 Institute of Anatomy and Anthropology, Riga Stradins University, Kronvalda Boulevard 9, LV-1010 Riga, Latvia; 2 Department of Oral and Maxillofacial Surgery and Oral Medicine, Riga Stradins University, 16 Dzirciema Street, LV-1007 Riga, Latvia; 3 Cleft Lip and Palate Centre, Institute of Stomatology, Riga Stradins University, 20 Dzirciema Street, LV-1007 Riga, Latvia

**Keywords:** cleft lip, cleft palate, cleft candidate genes, SHH, SOX3, WNT3A, WNT9B

## Abstract

Orofacial clefts have been associated with specific cleft candidate genes which encode regulatory proteins required for orofacial region development. Cleft candidate genes encode proteins involved with the cleft morphopathogenesis process, but their exact interactions and roles are relatively unclear in human cleft tissue. This study evaluates the presence and correlations of Sonic Hedgehog (SHH), SRY-Box Transcription Factor 3 (SOX3), Wingless-type Family Member 3A (WNT3A) and 9B (WNT9B) protein containing cells in different cleft tissue. Non-syndromic cleft-affected tissue was subdivided into three groups—unilateral cleft lip (UCL) (*n* = 36), bilateral cleft lip (BCL) (*n* = 13), cleft palate (CP) (*n* = 26). Control tissue was obtained from five individuals. Immunohistochemistry was implemented. The semi-quantitative method was used. Non-parametric statistical methods were applied. A significant decrease in SHH was found in BCL and CP tissue. SOX3, WNT3A and WNT9B had a significant decrease in all clefts. Statistically significant correlations were found. The significant decrease in SHH could be associated with BCL and CP pathogenesis. SOX3, WNT3A and WNT9B could have morphopathogenetic involvement in UCL, BCL, and CP. Similar correlations imply the presence of similar pathogenetic mechanisms in different cleft variations.

## 1. Introduction

Orofacial clefts are relatively common inborn anomalies with an average global prevalence of 1 per 700 live births [1,2]. Clefts of the orofacial region are described as developmental anomalies in which the fusion of developing facial folds and palate has been insufficient and defective, causing multiple possible types of facial clefts, including cleft lip with or without cleft palate [3,4].

The pathogenesis of non-syndromic orofacial clefts is complicated and involves multiple factors which could affect the facial cleft development process. Factors from the external environment which have been associated with the formation of orofacial clefts have been previously described and studied, including maternal health status, presence of alcohol abuse and smoking during pregnancy, nutritional status, presence of teratogens, such as retinoic acid, the use of specific medications, presence of infectious agents and other environmental factors [5]. Genetic factors also most likely play an important role in the cleft pathogenesis process by affecting and disturbing the correct facial region development process on the molecular level [6,7]. Cleft candidate genes, which mainly encode transcription factors and growth factors that affect the development of the orofacial region, have been previously associated with developmental abnormalities, such as cleft lip and palate [6,8]. Many of these genes regulate cell proliferation, cell adhesion and migration within the orofacial region tissue [5]. Genome-wide association studies (GWAS) have identified multiple gene polymorphisms associated with non-syndromic cleft lip and palate, with some variation being present in different populations [5,7,8]. Studies using next-generation sequencing (NGS) have been employed to uncover rarer coding variants of candidate genes associated with orofacial cleft etiology [9,10].

Different classification systems have been previously devised to categorize the variations of orofacial clefts based on their anatomical location, phenotypical variations and patterns, clinical characteristics, and other specifics [11,12,13]. Clefts of the orofacial region can be categorized by different types based on the location, including unilateral cleft lip, bilateral cleft lip, cleft lip with or without cleft palate, isolated cleft palate, cleft of the alveolus, clefts of the primary palate, clefts of the secondary palate [11,12]. A cleft lip with or without a cleft palate is defined as a cleft that affects the upper lip and, depending on the severity, can also affect the alveolus, primary and secondary palate. The cleft palate only typically affects the secondary palate [11,12]. The face develops by the fusion of facial prominences. During the 6th week of embryonic development, the maxillary processes fuse together with the medial nasal processes, which form the lateral part of the upper lip. Failure of fusion typically causes the formation of cleft lip [11]. Palatogenesis starts from the 5th week of embryonic development and continues until the 12th week, during which the primary and secondary palate develops. The secondary palate forms from paired palatal shelves that fuse together in the midline in a zip-like manner during the 9th to 12th week of embryonic development. Failure of palatal shelve fusion typically causes cleft palate [11]. During development, the newly formed craniofacial tissue has structural and regulatory differences in the lip and the palatal region, where the disruption could be mediated by separate pathogenetic mechanisms during cleft formation. Different cell groups interact during the development of the lip and palate, including ectodermal epithelial tissue, which is involved with the fusion process of facial prominences, as well as cranial neural crest cells, which migrate and proliferate throughout the facial prominences to form the soft connective tissue and bone tissue of the facial region [3,6]. In the case of developmental dysfunction, some cleft candidate genes and their coded regulatory factors might cause improper tissue growth and remodeling process within the developing orofacial region. These regulation differences based on the type of orofacial tissue could be different on the molecular level due to different regulatory roles within the developing lip or the developing palatal region [14,15,16]. Understanding these molecular differences, especially in cases of non-syndromic orofacial clefts, could be crucial for improving the understanding of cleft pathogenesis mechanisms and might provide new advances in the treatment and possible prevention of orofacial clefts.

Sonic Hedgehog (SHH) protein regulates cranial neural crest cell development and is involved in the development of cranial microvasculature [17]. SHH has been described as an important regulatory factor for upper jaw development by regulating the patterned growth process of this orofacial region [18]. SHH dysfunction has been associated with multiple embryonic malformations, including cleft lip and palate [19].

Sex-determining Region Y-Box Transcription Factor 3 (SOX3) is a protein that, with other Sex-determining Region Y-Box (SOX) proteins, regulates the development of neural plate, cranial neural crest cells of the craniofacial region [20]. SOX3 involvement has been previously described in craniofacial pathologies, including parathyroid gland aplasia [21], pituitary hypoplasia [22], persistent craniopharyngeal canal and a possible association with orofacial cleft formation [23]. 

Wingless-type Family Member 3A (WNT3A) protein regulates mesenchymal stem cell differentiation into osteoblasts [24] and cell proliferation [25] and provides regulation of facial mesenchyme development [26]. WNT3A has been associated with the formation of craniofacial clefts in human populations [27].

Wingless-type Family Member 9B (WNT9B) protein involvement with the development of the orofacial region, including the upper jaw and the lip, has been previously described in mice by regulating the growth, differentiation, and proliferation of mesenchymal cells within these regions [28,29]. WNT9B dysfunction has been associated with orofacial cleft formation in mice [30]. WNT9B has been associated with cleft formation in the human population [31,32].

Interactions between SHH, SOX3, WNT3A and WNT9B have been previously described. SOX3 interacts with β-catenin, which is an important signaling molecule in the canonical wingless-type (WNT) signaling pathway while also affecting WNT target genes, typically inducing downregulation of WNT signaling [33,34]. SOX3 has been described as a dose-dependent regulator of SHH transcription during craniofacial development [35]. WNT3A also interacts with SHH during craniofacial development [26], and WNT9B also can indirectly interact with Sonic Hedgehog (SHH) signaling during orofacial region development through WNT signaling [30].

This specific combination of immunohistochemically determined factors in multiple non-syndromic orofacial cleft types has not been previously analyzed in preceding studies in human tissue. SHH, SOX3, WNT3A and WNT3B proteins interact with each other through an interconnected network of signaling pathways, including SHH signaling and WNT signaling during the craniofacial development process. SHH, SOX3, WNT3A and WNT9B proteins have been designated for this research due to their previously chronicled involvement with the development of orofacial clefts and to better acknowledge their appearance in cleft-affected tissue, which could possibly establish their involvement and locational differences within different cleft affected tissue type variations.

The main aim of this specific study is to assess the presence of SHH, SOX3, WNT3A and WNT9B proteins by immunohistochemistry within the non-syndromic cleft-affected epithelium and connective tissue in non-syndromic orofacial cleft patient groups together with a comparison with the control group. Another important aim of this study is to assess correlations between the evaluated proteins to provide information about interactions between them in cleft tissue.

## 2. Materials and Methods

### 2.1. Characterization of Patient Groups and Tissue Material

Every tissue sample from the patient groups and control group which were examined in the study was provided as a donation with an elective agreement from the parents and/or legal guardians of cleft patients and the parents. The tissue material of patient groups was attained in the Cleft Lip and Palate Centre of the Institute of Stomatology of Riga Stradins University. The examination of patient and control group tissues was accomplished in the Riga Stradins University Department of Morphology. The Riga Stradins University Ethics Committee issued permissions for the acceptance and implementation of the study protocol (the first approval was dated 22 May 2003; the second approval Nr. 6-1/10/11 dated 24 September 2020). This research was coordinated following the regulations set in the Declaration of Helsinki, which was established in 1964.

Patient groups were subdivided into unilateral cleft lip (UCL), bilateral cleft lip (BCL) and cleft palate (CP) tissue groups obtained from cleft-correcting surgery. Patients from which cleft tissue was obtained had the diagnosis of cheilognatouranoschisis present. Cleft lip tissue was separated into unilateral cleft lip and bilateral cleft lip tissue because the bilateral cleft lip is typically described as a more serious phenotype clinically, and there might be morphopathogenetic differences in this tissue in comparison to unilateral cleft lip tissue. The soft tissue obtained from cleft palate correcting surgeries was assembled in a separate group for morphological evaluation. The tissue material attained from cleft-correcting surgery from each patient contained oral cavity epithelium and connective tissue. 

The inclusion criteria for cleft patient groups were the following: a diagnosis of orofacial cleft (UCL, BCL, CP), cleft-correcting surgery had been performed to treat said craniofacial cleft, no inflammatory or traumatic craniofacial pathology in anamnesis, no other congenital pathology found on clinical evaluation, patient age during surgery defined from 3 to 18 months before and during primary dentition age. In total, tissue material was received from 36 UCL patients, from which there were 20 boys and 16 girls aged 3–18 months, 13 BCL patients from which there were 10 boys and 3 girls aged 4–16 months and 26 CP patients from which there were 18 boys and 8 girls aged 4–14 months.

Control group tissue material was obtained from the upper lip tissue of 5 individuals (4 newborns and one fetus in the 24th week of gestation) not affected by craniofacial clefts and with age before the eruption of primary dentition taken from the historical collection of the Institute of Anatomy and Anthropology of Riga Stradins University. Control group individuals were newborns with specific causes of death not related to cleft lip and palate (2 newborns were affected with asphyxia due to umbilical cord compression, 2 newborns were affected with sudden infant death syndrome and 1 individual who was 24 weeks old was spontaneously aborted due to problematic maternal health status). The criteria for inclusion within the control group were no craniofacial clefts found on examination, no craniofacial clefts in patient anamnesis and family history, no other pathological process found in oral cavity tissue material, for example, inflammation, no other congenital abnormalities or other pathologies found during clinical investigation. The approval Nr. 2-PĒK-4/492/2022 for the use of control tissue was issued on 21 November 2022.

Anatomical localization from which patient and control group tissue was taken is illustrated in Supplementary Materials S1.

### 2.2. Immunohistochemistry and Slide Evaluation

Tissue specimens were prepared for microscopy and immunohistochemical evaluation by utilizing standard streptavidin and biotin immunostaining methods [36] for the identification of SHH, SOX3, WNT3A and WNT9B protein-containing cells in patient and control tissue. The tissue material was set in 2% formaldehyde and 0.2% picric acid within 0.1 M phosphate buffer solution for fixation. Phosphate buffer and saline solution, together with 10% saccharose, were applied for the washing procedure for no less than 12 h. Afterward, tissue specimens were immersed within paraffin, and later, the paraffinized blocks containing tissue material were sliced into several sections, with each section being 6–7 μm thin. Deparaffinization of previously mentioned tissue sections was implemented, and biotin-streptavidin immunostaining method was employed to notify the presence of previously mentioned factors in the tissue material with antibodies for Sonic Hedgehog (LS-C49806, 1:100, LifeSpan BioSciences, Inc., Seattle, WA, USA), Sex-determining Region Y-box Transcription Factor 3 (orb158460, 1:100, Biorbyt Ltd., Cambridge, UK), Wingless-type Family Member 3A (ab19925, 1:800, Abcam, Cambridge, UK), and Wingless-type Family Member 9B (ab151220, 1:100, Abcam, Cambridge, UK). Positive controls were performed for each antibody as set by manufacturer specifications. Negative controls were also performed where the primary antibody was excluded. Slide pictures were taken with a Leica DC 300F digital camera (Leica Microsystems Digital Imaging, Cambridge, UK). The obtained slide pictures were processed and evaluated with the Image Pro Plus program (Media Cybernetics, Inc., Rockville, MD, USA).

Semi-quantitative counting method [37,38,39] was implemented for the non-parametric assessment of the relative frequency of SHH, SOX3, WNT3A and WNT9B protein-containing cells in the oral cavity epithelium and connective tissue within slides. Light microscopy was employed to evaluate slides within 5 separate visual fields in each slide, and slide semi-quantitative assessment was performed separately by 2 independent scientists. Examples of semiquantitative evaluation can be found in Supplementary Materials S2. The following semi-quantitative designations [40] were implemented: 0—no factor-positive cells in the visual field (0%); 0/+—a rare occurrence of factor-positive cells in the visual field (0–12.5%); +—a few factor-positive cells in the visual field (12.5–25%); +/++—few to a moderate number of factor-positive cells in the visual field (25–37.5%); ++—a moderate number of factor-positive cells in the visual field (37.5–50%); ++/+++—moderate to a numerous number of factor-positive cells in the visual field (50–62.5%); +++—numerous factor positive cells in the visual field (62.5–75%); +++/++++—numerous to an abundant number of factor-positive cells in the visual field (75–87.5%); ++++—an abundant number of factor-positive cells in the visual field (87.5–100%).

### 2.3. Statistical Methods

Data analysis was achieved with the utilization of descriptive statistics and analytical, statistical methods. To evaluate the semiquantitative count of immunoreactive cells in each visual field, descriptive statistical methods, such as the calculation of the median within each group and the interquartile range, were implemented. The semiquantitative number of SHH, SOX3, WNT3A and WNT9B protein-containing cells has been given as a median for each group. The Spearman’s rho value (r_s_) for the strength of the correlation was comprehended as the following values: a very weak correlation was defined as r_s_ = 0.0–0.2, a weak correlation was defined as r_s_ = 0.2–0.4, a moderate correlation was defined as r_s_ = 0.4–0.6, a strong correlation was defined as r_s_ = 0.6–0.8 and a very strong correlation was defined as r_s_ = 0.8–1.0. The estimation of the statistical significance of the difference between the count of SHH, SOX3, WNT3A and WNT9B protein-containing cells within each patient group and control tissue was managed with the utilization of Kruskal–Wallis H test (H—Kruskal–Wallis H test statistic) for all groups and Mann–Whitney U test (U—Mann–Whitney U test statistic) for the comparison between cleft patient groups and controls. Statistical calculations were conducted by employing Statistical Product and Service Solutions (SPSS) Statistics version 25.0 (IBM Company, Chicago, IL, USA). The statistical significance for every statistical assessment was acknowledged with a *p*-value of < 0.05.

## 3. Results

### 3.1. SHH Immunohistochemistry

**Figure 1:**
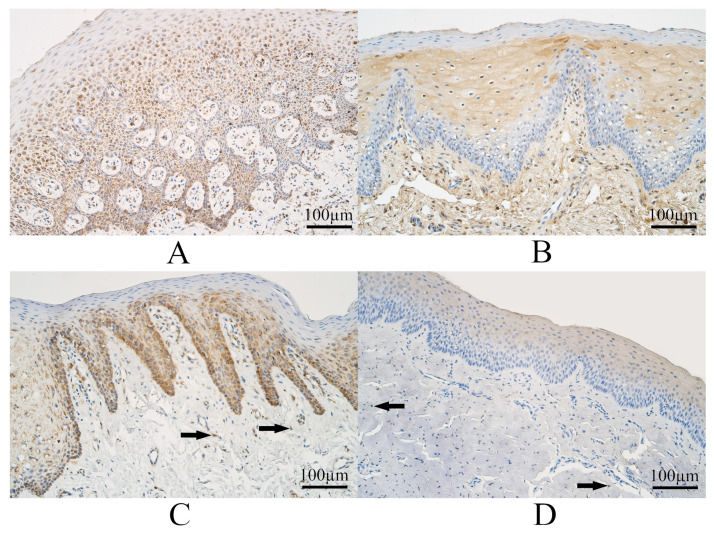
Sonic Hedgehog (SHH) immunohistochemistry within the control group and cleft-affected tissue. (**A**) Control with numerous SHH-containing epithelial cells and a moderate number of SHH immunoreactive cells in connective tissue, SHH immunohistochemistry, 200×. (**B**) Patient from unilateral cleft lip group with a moderate number of SHH-containing epithelial cells and connective tissue cells, SHH immunohistochemistry, 200×. (**C**) Patients from bilateral cleft lip group with moderate to numerous SHH immunoreactive epithelial cells and few to moderate SHH immunoreactive cells in connective tissue (arrows), SHH immunohistochemistry, 200×. (**D**) Patients from the cleft palate group with moderate to numerous SHH immunoreactive epithelial cells and with a few SHH immunoreactive cells in connective tissue (arrows), SHH immunohistochemistry, 200×.

The median number of SHH immunopositive epitheliocytes in controls was moderate to numerous and had a range from a moderate number of SHH immunoreactive cells to numerous SHH immunoreactive epitheliocytes. The control group connective tissue had a median number of SHH immunopositive structures moderate, and it varied from few to moderate immunopositive structures to a large number of SHH immunoreactive connective tissue cells (Figure 1A).

The median number of SHH-containing epitheliocytes in the tissue of UCL tissue was moderate to numerous and with a variation from few to moderate to numerous to abundant SHH immunoreactive epitheliocytes. In UCL tissue, the median number of SHH immunoreactive connective tissue cells was moderate to numerous and with a variation from a few to numerous to an abundant number of SHH immunoreactive cells (Figure 1B).

The median number of SHH immunoreactive surface epitheliocytes in the tissue of BCL was moderate, and it varied from no SHH-containing cells to numerous SHH-positive epitheliocytes. In BCL tissue, the median number of SHH immunopositive connective tissue cells was few to moderate and with a variation from a few to moderate to a numerous number of SHH immunoreactive connective tissue cells (Figure 1C).

The median number of SHH immunopositive epitheliocytes in CP tissue was few to moderate and with a variation from a few to a moderate number of SHH immunoreactive epitheliocytes. In CP connective tissue, the median number of SHH immunoreactive cells was few to moderate and with a variation from a few to a moderate number of SHH immunopositive connective tissue structures (Figure 1D).

Some examples of SHH immunohistochemistry in each group are visible in Figure 1.

Kruskal–Wallis H test revealed a statistically notable difference (H = 43.261, df = 3, *p* < 0.001) between cleft tissue groups together with controls in the number of SHH immunoreactive surface epithelial cells and a statistically significant difference (H = 40.626, df = 3, *p* < 0.001) was notified in the number of SHH immunoreactive connective tissue structures between cleft tissue groups and controls. 

The calculation of the Mann–Whitney U Test between the control group and cleft tissue groups indicated the following. No statistically notable difference (U = 85.5, *p* = 0.852) was found in the number of SHH immunoreactive epithelial cells between control tissue and UCL tissue. No statistically notable difference (U = 58.5, *p* = 0.189) was seen in the number of SHH immunoreactive connective tissue structures between control tissue and UCL tissue. A statistically notable difference (U = 10.0, *p* = 0.020) was identified in the number of SHH immunoreactive epithelial cells between control tissue and BCL tissue. No statistically notable dissimilarity (U = 16.5, *p* = 0.105) was found in the number of SHH immunoreactive connective tissue structures between the control tissue and the BCL tissue. A statistically notable difference (U = 5.0, *p* = 0.001) was calculated in the number of SHH immunoreactive epitheliocytes between control tissue and CP tissue. A statistically notable dissimilarity (U = 14.5, *p* = 0.004) was found in the number of SHH immunoreactive connective tissue structures between control tissue and CP tissue.

### 3.2. SOX3 Immunohistochemistry

**Figure 2:**
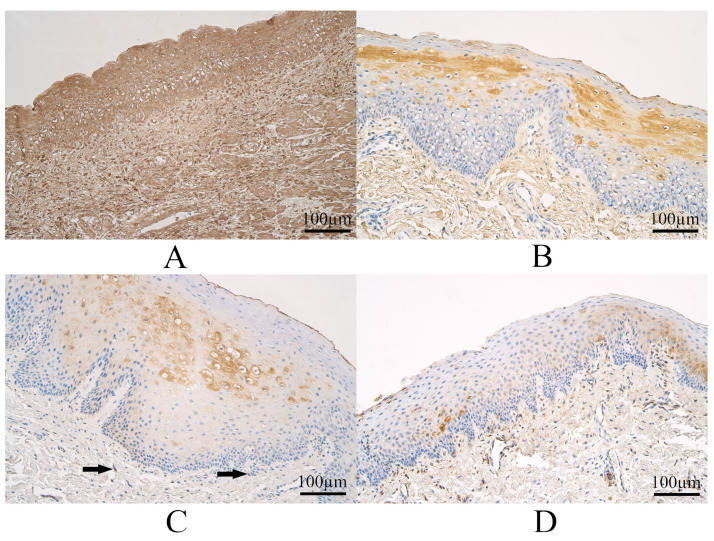
Sex-determining Region Y-box Transcription Factor 3 (SOX3) immunohistochemistry within the control group and cleft-affected tissue. (**A**) Control with numerous to abundant SOX3-containing epithelial and connective tissue cells, SOX3 immunohistochemistry, 200×. (**B**) Unilateral cleft lip patient group with a moderate number of SOX3-containing epitheliocytes and cells in connective tissue, SOX3 immunohistochemistry, 200×. (**C**) Patient from the bilateral cleft lip group with a moderate number of SOX3-containing epitheliocytes and a few SOX3 positive connective tissue cells (arrows), SOX3 immunohistochemistry, 200×. (**D**) Cleft palate patient with few to moderate SOX3-containing epitheliocytes and a moderate number of SOX3 positive cells in connective tissue, SOX3 immunohistochemistry, 200×.

The median number of SOX3 immunoreactive epitheliocytes in controls was numerous to abundant, and it varied from numerous to an abundant number of SOX3 immunopositive epitheliocytes. The median number of SOX3-containing connective tissue cells of controls was numerous, and it varied from moderate to numerous to a moderate number of SOX3 immunoreactive connective tissue cells (Figure 2A).

The median number of SOX3 immunopositive epitheliocytes in UCL tissue was moderate to numerous and with a variation from a few to numerous to abundant SOX3 positive epitheliocytes. The median number of SOX3 immunopositive connective tissue structures in UCL tissue was moderate and with a variation from a few to numerous SOX3 immunoreactive structures (Figure 2B).

The median number of SOX3 Immunoreactive surface epitheliocytes in BCL tissue was moderate and with a variation from a few to numerous SOX3-positive epitheliocytes. The median number of SOX3 immunopositive connective tissue cells in BCL tissue was moderate—moderate to numerous and with a variation from a few to numerous SOX3 positive connective tissue cells (Figure 2C).

The median number of SOX3 immunopositive epitheliocytes in CP tissue was few to moderate and with a range from a few to moderate to numerous SOX3 positive epitheliocytes. The median number of SOX3 immunopositive connective tissue cells in CP tissue was moderate and with a variation from a few to moderate to numerous SOX3 immunoreactive cells (Figure 2D).

Some examples of SOX3 immunohistochemistry in each group are visible in Figure 2.

The calculation of the Kruskal–Wallis H test displayed a statistically notable difference (H = 34.178, df = 3, *p* < 0.001) between cleft tissue and control groups in the number of SOX3 immunopositive epithelial cells and a statistically notable difference (H = 12.838, df = 3, *p* = 0.005) was calculated in the number of SOX3 immunoreactive connective tissue cells between cleft tissue and controls. 

The calculation of the Mann–Whitney U Test exhibited the following findings. A statistically notable difference (U = 14.5, *p* = 0.002) was calculated in the number of SOX3 immunopositive epithelial cells between control tissue and UCL tissue. A statistically significant difference (U = 10.0, *p* = 0.001) was seen in the number of SOX3 immunoreactive connective tissue cells between the controls and the UCL tissue group. A statistically notable difference (U = 1.0, *p* = 0.001) was identified in the number of SOX3 immunoreactive epithelial cells between the control tissue and BCL tissue group. A statistically notable difference (U = 4.5, *p* = 0.005) was revealed in the number of SOX3 immunoreactive connective tissue structures between control tissue and BCL tissue. A statistically notable difference (U = 0.0, *p* < 0.001) was found in the number of SOX3 immunoreactive epithelial cells between control tissue and CP tissue. A statistically notable difference (U = 4.0, *p* = 0.001) was identified in the number of SOX3 immunoreactive connective tissue structures between control tissue and CP tissue.

### 3.3. WNT3A Immunohistochemistry

**Figure 3:**
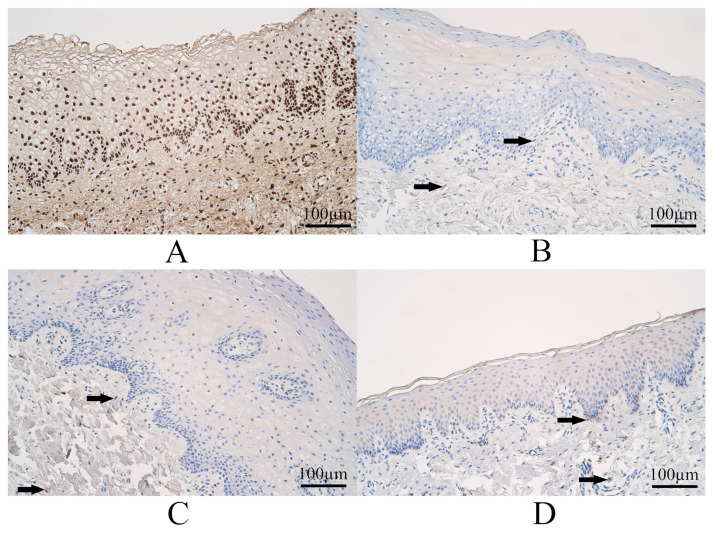
Wingless-type Family Member 3A (WNT3A) immunohistochemistry within controls and cleft-affected tissue. (**A**) Control with numerous WNT3A immunoreactive epithelial cells and cells in connective tissue, WNT3A immunohistochemistry, 200×. (**B**) Patients from the unilateral cleft lip group with few to moderate WNT3A immunoreactive epithelial cells and a few WNT3A immunoreactive cells in connective tissue (arrows), WNT3A immunohistochemistry, 200×. (**C**) Patients from the bilateral cleft lip group with a few WNT3A-containing surface epithelial cells and cells in connective tissue (arrows), WNT3A immunohistochemistry, 200×. (**D**) Patient from the cleft palate group with a moderate number of weakly stained WNT3A immunopositive epitheliocytes and with a few WNT3A immunoreactive connective tissue cells (arrows), WNT3A immunohistochemistry, 200×.

The median count of WNT3A immunoreactive epitheliocytes in controls was moderate to numerous, and it varied from moderate to numerous WNT3A positive surface epitheliocytes. In control connective tissue, the median number of WNT3A-containing connective tissue structures was numerous, and it ranged from moderate to numerous to an abundant number of WNT3A immunoreactive connective tissue cells (Figure 3A).

The median number of WNT3A immunoreactive surface epitheliocytes in UCL tissue was few to moderate, and it ranged from no positive cells to moderate to numerous WNT3A immunoreactive epitheliocytes. The median count of WNT3A positive connective tissue cells within the UCL group was a few, and it extended from no positive cells to a few to a moderate number of WNT3A immunoreactive connective tissue structures (Figure 3B).

The median number of WNT3A immunoreactive epitheliocytes in BCL tissue was a few, and it covered a range from no positive epitheliocytes to a moderate number of WNT3A immunoreactive epitheliocytes. The median count of WNT3A immunoreactive connective tissue cells in BCL was a few, and it varied from no immunoreactive connective tissue cells to a few to a moderate number of WNT3A immunoreactive cells (Figure 3C).

The median count of WNT3A immunoreactive epitheliocytes in CP tissue was a few, and it covered a range from no positive epithelial cells to a moderate number of WNT3A-containing epitheliocytes. The median count of WNT3A immunoreactive connective tissue cells in CP tissue was few to moderate and had a variation from no immunoreactive structures to few to a moderate number of WNT3A immunoreactive connective tissue cells (Figure 3D).

Some examples of WNT3A immunohistochemistry in each group are visible in Figure 3.

The calculation of the Kruskal–Wallis H test provided a statistically notable difference (H = 19.849, df = 3, *p* < 0.001) between cleft tissue and controls in the number of WNT3A immunoreactive epitheliocytes and a statistically notable difference (H = 16.259, df = 3, *p* = 0.001) was also present between cleft tissue and controls in the number of WNT3A immunoreactive connective tissue structures.

The calculation of the Mann–Whitney U Test identified the following information. A statistically notable (U = 19.0, *p* = 0.004) difference was calculated in the number of WNT3A immunoreactive epitheliocytes between control tissue and UCL tissue. A statistically notable difference (U = 0.0, *p* < 0.001) was identified in the number of WNT3A immunoreactive connective tissue cells between controls and the UCL tissue group. A statistically notable difference (U = 1.0, *p* = 0.001) was identified in the number of WNT3A immunoreactive epitheliocytes between controls and BCL tissue. A statistically notable difference (U = 0.0, *p* = 0.001) was revealed in the number of WNT3A immunoreactive connective tissue cells between controls and the BCL tissue group. A statistically notable difference (U = 1.0, *p* < 0.001) was indicated in the number of WNT3A immunoreactive epithelial cells between the controls and CP tissue group. A statistically notable difference (U = 0.0, *p* < 0.001) was identified in the number of WNT3A immunoreactive connective tissue cells between controls and CP tissue.

### 3.4. WNT9B Immunohistochemistry

**Figure 4:**
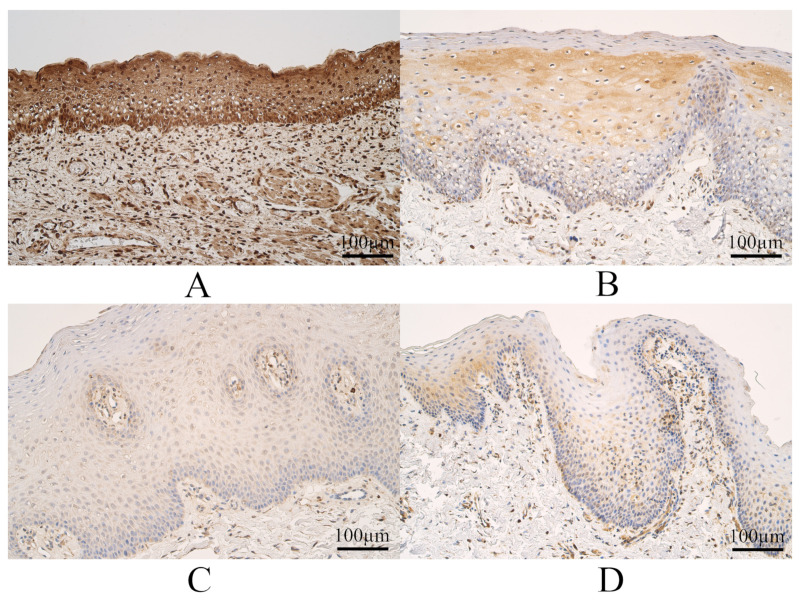
Wingless-type Family Member 9B (WNT9B) immunohistochemistry within controls and cleft-affected tissue. (**A**) Control with numerous to abundant WNT9B immunoreactive epitheliocytes and cells in connective tissue, WNT9B immunohistochemistry, 200×. (**B**) Patient from unilateral cleft lip group with moderate to numerous WNT9B-containing epitheliocytes and a moderate number of factor-positive cells in connective tissue, WNT9B immunohistochemistry, 200×. (**C**) Bilateral cleft lip patient with numerous WNT9B-containing epitheliocytes and moderate to numerous factor-positive connective tissue cells, WNT9B immunohistochemistry, 200×. (**D**) Cleft palate patient with a moderate number of WNT9B-containing epitheliocytes and moderate to numerous WNT9B positive connective tissue cells, WNT9B immunohistochemistry, 200×.

The median count of WNT9B immunoreactive epitheliocytes in controls was numerous to abundant and had a variation from numerous to an abundant number of WNT9B immunoreactive epitheliocytes. The median count of WNT9B containing connective tissue cells in controls was numerous to abundant, and it varied from numerous to an abundant number of WNT9B positive connective tissue cells (Figure 4A).

The median number of WNT9B positive surface epithelial cells in UCL tissue was moderate to numerous and had a variation from a few to numerous to abundant WNT9B immunoreactive epitheliocytes. The median count of WNT9B immunoreactive connective tissue cells within the UCL group was numerous, and it varied from moderate to numerous to an abundant number of WNT9B containing connective tissue structures (Figure 4B).

The median number of WNT9B immunoreactive epitheliocytes in BCL tissue was moderate to numerous and had a variation from a few to numerous WNT9B immunoreactive epitheliocytes. The median number of WNT9B-containing connective tissue cells in BCL was moderate to numerous and had a variation from a few too numerous to an abundant number of WNT9B-containing cells (Figure 4C).

The median number of WNT9B immunoreactive epitheliocytes in CP tissue was moderate and had a variation from a few to numerous numbers of WNT9 B-containing epitheliocytes. The median number of WNT9B immunoreactive connective tissue cells in CP tissue was moderate to numerous and had a variation from a few to numerous WNT9B immunoreactive cells (Figure 4D).

Some examples of WNT9B immunohistochemistry in each group are visible in Figure 4.

The calculation of the Kruskal–Wallis H test identified a statistically notable difference (H = 26.492, df = 3, *p* < 0.001) between cleft tissue and control groups in the number of WNT9B immunoreactive epithelial cells and a statistically notable difference (H = 20.123, df = 3, *p* < 0.001) was revealed in the number of WNT9B immunoreactive connective tissue structures between cleft tissue and control groups.

The calculation of the Mann–Whitney U Test identified the following information. A statistically notable difference (U = 14.0, *p* = 0.002) was identified in the number of WNT9B immunoreactive epithelial cells between control tissue and UCL tissue. A statistically notable difference (U = 32.5, *p* = 0.017) was also identified in the number of WNT9B immunoreactive connective tissue cells between controls and UCL tissue. A statistically notable difference (U = 0.5, *p* = 0.001) was determined in the number of WNT9B immunoreactive epithelial cells between the controls and the BCL group. A statistically notable difference (U = 4.5, *p* = 0.005) was identified in the number of WNT9B immunoreactive connective tissue cells between controls and BCL tissue. A statistically notable difference (U = 1.0, *p* < 0.001) was identified in the number of WNT9B immunoreactive epithelial cells between controls and CP tissue. A statistically notable difference (U = 2.5, *p* = 0.001) was calculated in the number of WNT9B immunoreactive connective tissue cells between control tissue and CP tissue.

SHH, SOX3, WNT3A and WNT9B protein immunoreactivity and the semiquantitative evaluation of these proteins are outlined in Table 1.

### 3.5. Correlations in Cleft-Affected Tissue

Determination of Spearman’s rank correlation coefficient enabled to discover of multiple statistically notable correlations between the number of SHH, SOX3, WNT3A and WNT9B protein immunoreactive cells in the epithelium and the connective tissue in each tissue group.

#### 3.5.1. Correlations in Controls

A statistically significant, very strong correlation (r_s_ = 0.8–1.0) was identified in controls between the number of SHH-containing epitheliocytes and the number of SHH-containing connective tissue cells (r_s_ = 0.892, *p* = 0.042).

#### 3.5.2. Correlations in UCL Tissue

A statistically notable strong correlation (r_s_ = 0.6–0.8) was calculated between the number of WNT9B immunoreactive epithelial cells and the number of WNT9B immunoreactive connective tissue structures (r_s_ = 0.662, *p* < 0.001).

Statistically notable moderate correlations (r_s_ = 0.4–0.6) were calculated with the number of factor positive cells—between SOX3 immunoreactive epithelial cells and SOX3 immunoreactive connective tissue structures (r_s_ = 0.599, *p* < 0.001), between SHH immunoreactive epithelial cells and WNT9B immunoreactive epitheliocytes (r_s_ = 0.578, *p* < 0.001), between SOX3 immunoreactive epitheliocytes and WNT9B immunoreactive epitheliocytes (r_s_ = 0.524, *p* = 0.001), between WNT3A immunoreactive epitheliocytes and WNT9B immunoreactive epitheliocytes (r_s_ = 0.521, *p* = 0.001), between SHH immunoreactive epithelial cells and WNT3A immunoreactive epithelial cells (r_s_ = 0.510, *p* = 0.001), between SOX3 immunoreactive epithelial cells and WNT3A immunoreactive epithelial cells (r_s_ = 0.496, *p* = 0.002), between SOX3 immunoreactive epithelial cells and WNT9B immunoreactive connective tissue cells (r_s_ = 0.445, *p* = 0.007), between SHH immunoreactive connective tissue cells and WNT3A immunoreactive epithelial cells (r_s_ = 0.444, *p* = 0.007), between SHH-containing connective tissue cells and SOX3-containing cells in connective tissue (r_s_ = 0.432, *p* = 0.008), between SHH immunoreactive epitheliocytes and SOX3 immunoreactive epithelial cells (r_s_ = 0.415, *p* = 0.012).

Statistically notable weak correlations (r_s_ = 0.2–0.4) were calculated with the number of protein-containing cells—between SOX3 immunoreactive connective tissue structures and WNT9B immunoreactive connective tissue structures (r_s_ = 0.293, *p* = 0.018), between SHH immunoreactive connective tissue structures and WNT3A immunoreactive connective tissue cells (r_s_ = 0.384, *p* = 0.021), between SOX3 immunoreactive connective tissue cells and of WNT3A immunoreactive epitheliocytes (r_s_ = 0.375, *p* = 0.024), between SHH immunoreactive epithelial cells and WNT9B immunoreactive connective tissue cells (r_s_ = 0.374, *p* = 0.025), between SHH immunoreactive connective tissue cells and SOX3 immunoreactive epithelial cells (r_s_ = 0.341, *p* = 0.042).

The summary of statistically notable correlations in the UCL tissue group can be seen in Table 2.

#### 3.5.3. Correlations in BCL Tissue

A statistically notable, very strong positive correlation (r_s_ = 0.8–1.0) was calculated between the number of SHH immunoreactive epithelial cells and WNT9B immunoreactive epithelial cells (r_s_ = 0.812, *p* = 0.001).

Statistically notable strong positive correlations (r_s_ = 0.6–0.8) were calculated with the number of protein immunoreactive cells—between SOX3 immunoreactive epithelial cells and WNT9B immunoreactive epithelial cells (r_s_ = 0.713, *p* = 0.009), between WNT3A immunoreactive epithelial cells and WNT9B immunoreactive connective tissue structures (r_s_ = 0.687, *p* = 0.014), between WNT3A immunoreactive epithelial cells and WNT9B immunoreactive epithelial cells (r_s_ = 0.0659, *p* = 0.020), between SHH immunoreactive connective tissue structures and SOX3 immunoreactive epithelial cells (r_s_ = 0.617, *p* = 0.032).

Statistically notable moderate correlations (r_s_ = 0.4–0.6) were determined. A correlation between the number of SHH immunoreactive connective tissue structures and WNT3A immunoreactive epithelial cells (r_s_ = 0.591, *p* = 0.043) was identified. Another correlation was found between SHH immunoreactive epithelial cells and SOX3 immunoreactive epithelial cells (r_s_ = 0.580, *p* = 0.048).

Statistically notable correlations in the BCL tissue group have been summarized in Table 3.

#### 3.5.4. Correlations in CP Tissue

A statistically notable, very strong positive correlation (r_s_ = 0.8–1.0) was noticed between the number of SOX3 immunoreactive epithelial cells and WNT9B immunoreactive epithelial cells (r_s_ = 0.832, *p* < 0.001).

A statistically notable moderate positive correlation (r_s_ = 0.4–0.6) was calculated between SHH immunoreactive connective tissue cells and SOX3 immunoreactive connective tissue cells (r_s_ = 0.440, *p* = 0.025).

A statistically significant weak positive correlation (r_s_ = 0.2–0.4) was identified between SOX3 immunoreactive connective tissue structures and WNT9B immunoreactive connective tissue structures (r_s_ = 0.397, *p* = 0.050).

Statistically notable moderate negative correlations (r_s_ = −0.6…−0.4) were calculated. A correlation was found between SOX3 immunoreactive connective tissue structures and WNT3A immunoreactive epithelial cells (r_s_ = −0.458, *p* = 0.028). Another correlation was calculated between the number of SHH immunoreactive epithelial cells and SOX3 immunoreactive connective tissue structures (r_s_ = −0.417, *p* = 0.043).

Statistically notable correlations in the CP tissue group are summarized in Table 4.

## 4. Discussion

### 4.1. Immunohistochemistry

Various statistically notable differences have been noted in the number of SHH, SOX3, WNT3A, and WNT9B immunoreactive cells between cleft tissue and controls.

Evaluation of SHH-containing cells indicated no statistically notable dissimilarities in the number of SHH immunoreactive epithelial cells and connective tissue structures between the controls and UCL tissue group. Statistically significant differences for SHH immunoreactivity were found in the epithelium between controls and the BCL tissue group showing a significant decrease of SHH immunoreactive cells but not within the underlying connective tissue. Statistically notable differences in SHH immunoreactivity were found between controls and the CP tissue group, both in surface epitheliocytes and connective tissue cells showing a significant decrease in the number of SHH immunoreactive cells in comparison to controls. SHH has been previously described as an important factor during lip development, and overexpression of the *SHH* gene in mice has shown malformations of the developing lip [41]. SHH signaling is needed for the closure of the upper lip by enhancing the proliferation of ectomesenchymal cells during lip development [42]. SHH signaling is also crucial during palate development, and disturbances of SHH function during epithelial-mesenchymal interactions typically result in CP formation [43]. Our results indicate that SHH might be more involved with CP and BCL than UCL in postnatal human tissue. The difference in SHH activity between the BCL and UCL epithelium could be a possible new direction for evaluation in future research to specify the possible differences in molecular regulatory mechanisms between these two types of clefts.

SOX3 immunoreactivity assessment identified statistically significant differences between controls and all evaluated cleft types (UCL, BCL, CP) in the number of SOX3 immunoreactive cells in the surface epithelium and the connective tissue showing a significant decrease for the number of SOX3 immunoreactive cells in comparison to controls. SOX3 has been previously described as a factor involved with the formation of the orofacial region structures, such as the origination of the pharyngeal pouches and the migration of neural crest cells within this body region [44]. The statistically significant differences found in all evaluated cleft tissue types indicate that SOX3 might be involved with the regulatory mechanisms of postnatal cleft lip and cleft palate tissue differentiation by possibly affecting tissue growth and proliferation locally in these regions. Previously SOX3 has been described as a regulatory factor expressed in the developing neural plate and nervous tissue [20], but possible SOX3 involvement in craniofacial cleft morphogenesis has not been widely described previously.

For WNT3A, statistically notable differences were calculated between control tissue and all three cleft tissue groups, both in the epithelium and the underlying connective tissue showing a significant decrease in the number of WNT3A immunoreactive cells in comparison to controls. WNT3A is a notable regulator of craniofacial region development through WNT signaling [26]. *WNT3A* gene dysfunction has been previously associated with the formation of cleft lip and palate in humans [45]. The results of our study show that the decreased number of WNT3A immunoreactive cells in all evaluated cleft-affected tissue groups compared to controls again emphasizes the role of WNT signaling during non-syndromic cleft lip and palate morphogenesis in human postnatal tissue.

WNT9B immunoreactivity evaluation revealed statistically significant variations between control tissue and all three cleft tissue types within the epithelium and the underlying connective tissue showing a significant decrease in the number of WNT9B immunoreactive cells in comparison to controls. *WNT9B* gene mutations and dysfunction in mice have been associated with the development of orofacial clefts, such as cleft lip and cleft palate, by disrupting the WNT signaling pathways within the developing orofacial tissue [31,32]. Our study results indicate that WNT9B could be involved with the morphogenesis of UCL, BCL and CP in postnatal human tissue by possibly affecting the WNT signaling pathway within the growing lip and palate tissue in humans. The larger number of WNT9A-containing cells in cleft tissue in comparison to the number of WNT3A-containing cells indicates some variations and differences in the possible morphogenetic role of these proteins during cleft tissue growth, which could be evaluated in future studies.

### 4.2. Correlations

#### 4.2.1. Control Tissue Correlations

A statistically notable, very strong correlation was identified between the number of SHH-containing epitheliocytes and SHH-containing connective tissue cells in controls. SHH is a notable regulatory factor during craniofacial development and is expressed normally within the developing oral cavity epithelium, as well as in the cranial neural crest cell-derived ectomesenchymal tissue [17,46]. This specific correlation described in the control tissue probably could be an example of the active role of SHH during the growth and formation of normal craniofacial tissue.

Because there was a very limited number of control patients, the correlation found in this group could possibly be accidental due to the limited amount of available control tissue and must be analyzed with caution.

#### 4.2.2. UCL Tissue Correlations

The statistically notable strong correlation between the number of WNT9B-containing epithelial cells and WNT9B-containing connective tissue cells probably could indicate a strong activity of WNT signaling within the lip tissue where the importance of WNT9B interactions regarding cleft development has been notified [29,47]. This could be a compensatory regulatory mechanism in postnatal UCL tissue and could affect tissue growth and remodeling process in this location.

Multiple statistically significant moderate correlations were identified. The moderate correlation between SOX3-containing epitheliocytes and SOX3-containing connective tissue cells could indicate that SOX3 could provide some growth and tissue differentiation regulation both in the epithelium and connective tissue cells postnatal UCL tissue. SOX3 regulatory role during craniofacial region growth and development has been previously identified [44], and this correlation, together with the significant presence of SOX3-containing cells in UCL tissue in comparison to controls, could indicate a role of SOX3 activity during cleft lip postnatal morphogenesis.

Many of the evaluated moderate correlations involving WNT9B were identified within the epithelium of UCL tissue. The moderate correlations between the number of WNT9B-containing epitheliocytes and SHH-containing epitheliocytes, between WNT9B-containing epitheliocytes and SOX3-containing epitheliocytes, between WNT9B-containing epitheliocytes and WNT3A containing epitheliocytes could be understood that the cleft-affected oral epithelium is an important location for WNT9B interactions postnatal UCL tissue. The importance of WNT9B regulatory function through classical WNT signaling pathway has been demonstrated in mice [28,48], where the disruption of WNT9B function has been associated with the formation of cleft lip and palate. Interaction between WNT9B and SHH has been previously notified during lip and palate formation and growth process, where it has been suggested that a negative-feedback loop could be present between classical WNT signaling and SHH signaling pathways while non-canonical WNT signaling might have an activating effect on SHH signaling [30]. Our results could indicate that these complex interactions between WNT9B and SHH could be present in human postnatal UCL tissue, which could affect tissue growth and the remodeling process. The other interaction between classical WNT signaling and SOX3 has been notified previously [49], which could imply that WNT9B and SOX3 interactions could play a role during the growth of cleft-affected epithelium. The moderate correlation between WNT9B and WNT3A-containing cells could indicate a significant WNT signaling activity in the cleft-affected epithelium. WNT signaling is essential for the regulation of facial fold fusion during the development of the lip [50]. Specifically for postnatal UCL tissue, disturbed WNT signaling in the epithelium could probably continue to affect cleft tissue growth and maturation after birth. 

Some of the evaluated moderate correlations involving WNT3A were found between the factors in the epithelium. The correlations between WNT3A-containing epitheliocytes and SHH-containing epitheliocytes, between WNT3A-containing epitheliocytes and SOX3-containing epitheliocytes show that WNT3A could also take part during facial tissue growth and maturation process. WNT3A can affect SHH expression in the frontonasal ectodermal zone during early facial development [26], and the results of our study, in turn, could imply that WNT3A and SHH interactions could appear in cleft-affected tissue postnatally. The role of WNT3A and WNT signaling on SOX3 regulation has been notified in animal models [49,51], where increased WNT signaling activity could affect SOX3 function by regulating the induction process of neural crest cells while our results could show that some interactions between WNT3A and SOX3 could be present in the postnatal cleft-affected epithelium as well.

Some moderate correlations were found between the number of factor-positive cells in the surface epithelium and the connective tissue cells in UCL tissue. The moderate correlation between SOX3-containing epitheliocytes and WNT9B-containing connective tissue cells could also imply that interactions between WNT9B and SOX3 are present not only in the postnatal cleft-affected epithelium but also in the underlying connective tissue where WNT signaling together with SOX3 and other related factors could affect the regulation of oral cavity tissue growth and differentiation [48,49]. The moderate correlation between SHH-containing connective tissue cells and WNT3A-containing epitheliocytes could show that WNT and SHH signaling interactions could be present between underlying connective tissue and the surface epithelium for the postnatal UCL tissue specifically. The interactions between WNT signaling and SHH signaling have been previously described during palatogenesis [30] and during the early facial development process [26], while the information regarding interactions in postnatal human cleft tissue has not been described in detail previously.

The moderate correlation between SHH-containing connective tissue cells and SOX3-containing connective tissue cells could be informative in understanding how SOX3 and SHH could affect oral cavity connective tissue growth and formation postnatally. Previously interactions between SOX3 and SHH have been noted during forebrain development [35,52], but the exact interactions of SHH signaling and SOX3 in postnatal cleft-affected tissue are relatively unclear, but it might be speculated that SOX3 and SHH activity might be affected by interconnective action of WNT signaling on both SOX3 [49] and SHH [26,30] separately or possibly by some other mechanisms. The similar moderate correlation between SHH-containing epitheliocytes and SOX3-containing epitheliocytes could also imply that while SHH and SOX3 might have a more direct interaction during central nervous system development [35,52], the interactions of SHH and SOX3 in postnatal cleft-affected tissue could be affected and modified by other regulatory factors which might modify both SHH and SOX3.

Multiple statistically significant weak correlations were also identified within the UCL group. Some weak correlations involving SOX3 were found. The correlations between SOX3-containing connective tissue cells and WNT9B-containing connective tissue cells and the correlation between SOX3-containing connective tissue cells and WNT3A-containing epitheliocytes corresponds with previous knowledge about the possible interconnection of SOX3 and classical WNT signaling pathway [49]. Some weak correlations involving SHH were uncovered in UCL tissue. The correlation between SHH-containing connective tissue cells and WNT3A-containing connective tissue cells and the correlation between SHH-containing epitheliocytes and WNT9B-containing connective tissue cells implies that WNT and SHH signaling interactions and disturbances are probably important during the growth and morphogenesis of postnatal UCL tissue [30] which corresponds with the previously found similar moderate correlations between SHH and WNT3A or WNT9B in our research. The correlation between SHH-containing connective tissue cells and SOX3-containing epitheliocytes could again imply the presence of interconnecting regulatory mechanisms possibly mediated by WNT signaling in postnatal cleft-affected tissue or by some other regulatory factors.

#### 4.2.3. BCL Tissue Correlations

A statistically significant, very strong correlation was noted between SHH-containing epitheliocytes and WNT9B-containing epitheliocytes which emphasizes the role of WNT and SHH signaling within the postnatal BCL cleft-affected epithelium. In previous studies, SHH signaling disturbances and loss of SHH function in the developing and growing lip epithelium have been associated with both UCL and BCL [19,42], while WNT9B functional disturbances have been associated with cleft lip and palate in mice [53].

Multiple statistically significant strong positive correlations were identified. The strong correlation between SOX3-containing epitheliocytes and WNT9B-containing epitheliocytes in postnatal BCL tissue could be explained by SOX3 and WNT signaling interaction through β-catenin [49]. A similar statistically significant moderate correlation was also found in postnatal UCL tissue, which could imply a similarity between possible morphogenetic mechanisms in both cleft lip types postnatally. 

The strong correlation between WNT3A-containing epitheliocytes and WNT9B-containing connective tissue cells and the correlation between WNT3A-containing epitheliocytes and WNT9B-containing epitheliocytes are similar to the moderate correlations involving WNT3A and WNT9B seen in UCL tissue, and this also could possibly emphasize the significance of WNT3A and WNT9B activity within both types of postnatal cleft lip tissue. Interestingly both WNT3A and WNT9B are regulated by Pre-B-Cell Leukemia Homeobox (PBX) proteins which directly affect Wingless-type signaling and afterward moderate cell apoptosis during craniofacial development [54]. The possibility that some other factors could affect WNT3A and WNT9B function within postnatal cleft-affected tissue needs to be considered while trying to understand the complicated molecular interactions between these proteins.

The statistically significant strong correlation between SHH-containing connective tissue cells and SOX3-containing epithelial cells could be described by understanding the previously known roles of SHH and SOX3 during craniofacial region growth and morphogenesis. SHH regulates ectomesenchyme and surface epithelium growth and development in the craniofacial region [55], while SOX3, together with other factors, affects the origination and growth of pharyngeal pouches [54]. It might be possible that SHH could interact with SOX3 directly or indirectly within the postnatal oral cavity tissue as well. While this correlation was weak in UCL tissue, these similar correlations again could imply comparable morphogenetic mechanisms in both cleft lip types. The statistically significant moderate correlation was also notified between SHH-containing epitheliocytes and SOX3-containing epitheliocytes which might be interpreted in a similar manner to the previously mentioned correlation.

The statistically significant moderate correlation between SHH-containing connective tissue cells and WNT3A-containing epitheliocytes in postnatal BCL tissue, which was also identified in the UCL tissue, again emphasizes the possible similarities between both cleft lip types while regarding the interactions of SHH and WNT signaling within the lip tissue because both SHH signaling and WNT signaling are tightly interconnected during orofacial development and growth process [30].

#### 4.2.4. CP Tissue Correlations

The statistically significant, very strong correlation between SOX3-containing epitheliocytes and WNT9B-containing epitheliocytes could again be explained by the classical WNT signaling pathway and β-catenin binding to SOX3 which could impact the function of SOX3 within postnatal palatal tissue [49]. WNT9B dysfunction has previously been more associated with the formation of CP in mice [30] and in humans [31,32]. A similar correlation was also noticed in UCL and BCL tissue which could possibly imply similarities of morphogenetic mechanisms in all three postnatal cleft-affected tissue types.

The moderate correlation between SHH-containing connective tissue cells and SOX3-containing connective tissue cells could imply that SHH and SOX3 interactions could be a part of multiple regulatory processes which affect palatal connective tissue growth and cell differentiation. The role of SHH during palatogenesis has been previously notified [56], while SOX3 function within the palatal tissue is relatively unclear with a possible SOX3 interaction during pharyngeal pouch development [39] while the role of these proteins postnatally has not been well described in cleft tissue.

The weak correlation between SOX3-containing connective tissue cells and WNT9B containing connective tissue cells shows that classical WNT signaling pathway could interact with SOX3 in the postnatal cleft-affected palatal connective tissue [49], as well as in the epithelium. 

Interestingly some statistically significant negative correlations were found in the postnatal CP tissue involving SOX3-containing connective tissue cells. The statistically significant moderate negative correlation between SOX3-containing connective tissue cells and WNT3A-containing epitheliocytes could indicate a possible inhibitory action in the epithelium and the connective tissue between WNT3A and SOX3. WNT3A, together with other factors, could possibly provide inhibitory action on *SOX3* gene expression, which has been previously demonstrated in animal models [57]. The statistically significant moderate negative correlation between SOX3-containing connective tissue cells and SHH-containing epitheliocytes could mean that some inhibitory interaction could also be present between SOX3 and SHH in postnatal CP tissue. Complicated interactions between SHH and SOX3 have been demonstrated during brain development, whereas antagonistic interactions between SHH and SOX3 have been demonstrated during the formation of diencephalons in mice [52]. The exact role of this interaction in postnatal CP tissue remains uncertain, but the possibility that interactions with other craniofacial development and growth factors might assist in explaining this unique correlation.

### 4.3. Limitations of the Study

The main limitation of our study is the use of only immunohistochemistry for the assessment of SHH, SOX3, WNT3A and WNT9B proteins in various tissue groups. The implementation of other study techniques, including genetic studies or in situ hybridization, could provide more detailed and specific information regarding this type of research. The semi-quantitative method is just one of the possible methods which could be used to evaluate direct visualization of immunoreactive structures in tissue immunohistochemistry. This method cannot evaluate the concentration of proteins in the tissue, and additional methods like radio immunohistochemistry and enzyme-linked immunosorbent assay (ELISA) could provide a more quantitative assessment of each evaluated protein. The implementation of additional methods is planned in future studies analyzing cleft candidate genes and their coded proteins.

Although there are datasets and studies which have used animal models to assess cleft candidate genes, direct comparisons between human tissue and animal tissue would be problematic due to the differences in the number and types of regulatory genes, as well as differences in the timing of gene activation and expression between species. These comparisons could, unfortunately, lead to misleading conclusions, which could have a negative impact on understanding the delicate and complicated pathogenetic mechanisms of orofacial cleft formation in humans.

The lack of information regarding cleft candidate gene expression in human cleft-affected tissue complicates the evaluation of cleft morphopathogenesis. Results from animal studies about cleft pathogenesis are not always applicable to humans but are necessary to describe the known functions and actions of different cleft candidate genes. Information from animal studies at least provides an understanding of the prevalent regulatory mechanisms which might be involved in human orofacial cleft pathogenesis, but this information has to be understood with caution.

An important limitation to note would be the very small number of control group individuals which, unfortunately, could impact our research results. The ethical concerns surrounding the access to oral cavity tissue not affected by orofacial clefts raise difficulties with the availability of usable control tissue. Access to tissue from relatively healthy children not affected by clefts is very limited due to the lack of significant surgical indications to retrieve any relatively normal oral cavity tissue. The control tissue acquired from the historical collection of the Institute of Anatomy and Anthropology of Riga Stradins University is not exactly in the same age range as the patient group tissue, but it was the closest possible option available for the analysis and comparison with cleft tissue groups. It is very complicated to acquire any healthy oral cavity tissue without any pathological changes from a notable number of children of the same age as patient groups because of the ethical aspects and the necessity of specific indications regarding access to any tissue from children.

## 5. Conclusions

1. The statistically significant decrease of SHH in bilateral cleft lip connective tissue and cleft palate tissue could imply that SHH dysfunction might be more associated with the morphogenesis and tissue growth of these specific types of clefts postnatally and less with unilateral cleft lip.
2. The statistically significant decrease of SOX3, WNT3A and WNT9B proteins in all evaluated non-syndromic cleft-affected tissue types could indicate similarities in cleft morphogenesis and growth mechanisms within the postnatal cleft lip and palate tissue, possibly due to interlinked signaling pathway disruption, such as disrupted WNT signaling.
3. The presence of similar correlations between SHH, SOX3, WNT3A, and WNT9B protein-containing cells within different cleft-affected tissue types could indicate the existence of similar signaling mechanisms and interactions within each separate cleft type postnatally, while the possible differences in correlations between cleft types might be affected by other regulatory factors mediating and modifying SHH, SOX3, WNT3A, and WNT9B function.

## Figures and Tables

**Table 1:** Median values of semiquantitative evaluation for Sonic Hedgehog, Sex-determining Region Y-box Transcription Factor 3, Wingless-type Family Member 3A and Wingless-type Family Member 9B immunoreactivity within the controls and cleft-affected tissue groups.

	SHH		SOX3		WNT3A		WNT9B	
	Epithelium	Connective Tissue	Epithelium	Connective Tissue	Epithelium	Connective Tissue	Epithelium	Connective Tissue
Controls	++/+++	++	+++/++++	+++	++/+++	+++	+++/++++	+++/++++
Unilateral Cleft Lip	++/+++	++/+++	++/+++	++	+/++	+	++/+++	+++
Bilateral Cleft Lip	++	+/++	++	++–++/+++	+	+	++/+++	++/+++
Cleft Palate	+/++	+/++	+/++	++	+	+/++	++	++/+++
H	43.261	40.626	34.178	12.838	19.849	16.259	26.492	20.123
*p*	<0.001	<0.001	<0.001	0.005	<0.001	0.001	<0.001	<0.001

Abbreviations: SHH—Sonic Hedgehog; SOX3—Sex-determining Region Y-box Transcription Factor 3; WNT3A—Wingless-type Family Member 3A; WNT9B—Wingless-type Family Member 9B; E—epithelium; CT—connective tissue; H—Kruskal–Wallis H test statistic; *p*—*p*-value; 0/+—a rare occurrence of factor positive cells in the visual field; +—a few factor-positive cells in the visual field; +/++—few to a moderate number of factor-positive cells in the visual field; ++—a moderate number of factor-positive cells in the visual field; ++/+++—moderate to a numerous number of factor-positive cells in the visual field; +++—numerous factor positive cells in the visual field; +++/++++—numerous to an abundant number of factor-positive cells in the visual field; ++++—an abundant number of factor-positive cells in the visual field.

**Table 2:** Statistically notable correlations in the UCL group.

Strength of Correlation	Correlations between Factors in UCL Group	r_s_	*p*
Strong (0.6–0.8)	WNT9B in epithelium and WNT9B in connective tissue	0.662	<0.001
Moderate (0.4–0.6)	SOX3 in epithelium and SOX3 in connective tissue	0.599	<0.001
	SHH in epithelium and WNT9B in epithelium	0.578	<0.001
	SOX3 in epithelium and WNT9B in epithelium	0.524	0.001
	WNT3A in epithelium and WNT9B in epithelium	0.521	0.001
	SHH in epithelium and WNT3A epithelium	0.510	0.001
	SOX3 in epithelium and WNT3A in epithelium	0.496	0.002
	SOX3 in epithelium and WNT9B in connective tissue	0.445	0.007
	SHH in connective tissue and WNT3A in epithelium	0.444	0.007
	SHH in connective tissue and SOX3 in connective tissue	0.432	0.008
	SHH in epithelium and SOX3 in epithelium	0.415	0.012
Weak (0.2–0.4)	SOX3 in connective tissue and WNT9B in connective tissue	0.392	0.018
	SHH in connective tissue and WNT3A in connective tissue	0.384	0.021
	SOX3 in connective tissue and WNT3A in epithelium	0.375	0.024
	SHH in epithelium and WNT9B in connective tissue	0.374	0.025
	SHH in connective tissue and SOX3 in epithelium	0.341	0.042

Abbreviations: UCL—unilateral cleft lip; SHH—Sonic Hedgehog; SOX3—Sex-determining Region Y-box Transcription Factor 3; WNT3A—Wingless-type Family Member 3A; WNT9B—Wingless-type Family Member 9B; r_s_—Spearman’s rho value; *p*—*p*-value.

**Table 3:** Statistically notable correlations in the BCL group.

Strength of Correlation	Correlations between Factors in BCL Group	r_s_	*p*
Very strong (0.8–1.0)	SHH in epithelium and WNT9B in epithelium	0.812	0.001
Strong (0.6–0.8)	SOX3 in epithelium and WNT9B in epithelium	0.713	0.009
	WNT3A in epithelium and WNT9B in connective tissue	0.687	0.014
	WNT3A in epithelium and WNT9B in epithelium	0.659	0.020
	SHH in connective tissue and SOX3 in epithelium	0.617	0.032
Moderate (0.4–0.6)	SHH in connective tissue and WNT3A in epithelium	0.591	0.043
	SHH in epithelium and SOX3 in epithelium	0.580	0.048

Abbreviations: BCL—bilateral cleft lip; SHH—Sonic Hedgehog; SOX3—Sex-determining Region Y-box Transcription Factor 3; WNT3A—Wingless-type Family Member 3A; WNT9B—Wingless-type Family Member 9B; r_s_—Spearman’s rho value; *p*—*p*-value.

**Table 4:** Statistically notable correlations in the CP group.

Strength of Correlation	Correlations between Factors in CP Group	r_s_	*p*
Positive correlations			
Very strong (0.8–1.0)	SOX3 in epithelium and WNT9B in epithelium	0.832	<0.001
Moderate (0.4–0.6)	SHH in connective tissue and SOX3 in connective tissue	0.440	0.025
Weak (0.2–0.4)	SOX3 in connective tissue and WNT9B in connective tissue	0.397	0.050
Negative correlations			
Moderate (−0.6…−0.4)	SOX3 in connective tissue and WNT3A in epithelium	−0.458	0.028
	SHH in epithelium and SOX3 in connective tissue	−0.417	0.043

Abbreviations: CP—cleft palate; SHH—Sonic Hedgehog; SOX3—Sex-determining Region Y-box Transcription Factor 3; WNT3A—Wingless-type Family Member 3; WNT9B—Wingless-type Family Member 9B; r_s_—Spearman’s rho value; *p*—*p*-value.

## Data Availability

Due to ethical concerns surrounding the use of tissue material from children, the data are not publicly available. The data provided and analyzed in this research are available at the request of the corresponding author.

## Supplementary Materials

The following supporting information can be downloaded at: https://www.mdpi.com/article/10.3390/dj11060151/s1, Figure S1: Localization of tissue used for the immunohistochemical evaluation of cleft affected tissue (arrows).; Figure S2: Examples of semiquantitative evaluation of SHH, SOX3, WNT3A and WNT9B immunohistochemistry of cleft affected tissue and control tissue within 5 visual fields.
