# Supplementary material for: Characterization of SHH, SOX3, WNT3A and WNT9B Proteins in Human Non-Syndromic Cleft Lip and Palate Tissue
Source: Dent J (Basel). 2023 Jun 9;11(6):151. doi: 10.3390/dj11060151 (PMC10297181; doi:10.3390/dj11060151)
Supplement: Supplementary file 1 [file dentistry-11-00151-s001.zip › Supplementary material 1_Tissue localization.pdf]

Supplementary material 1

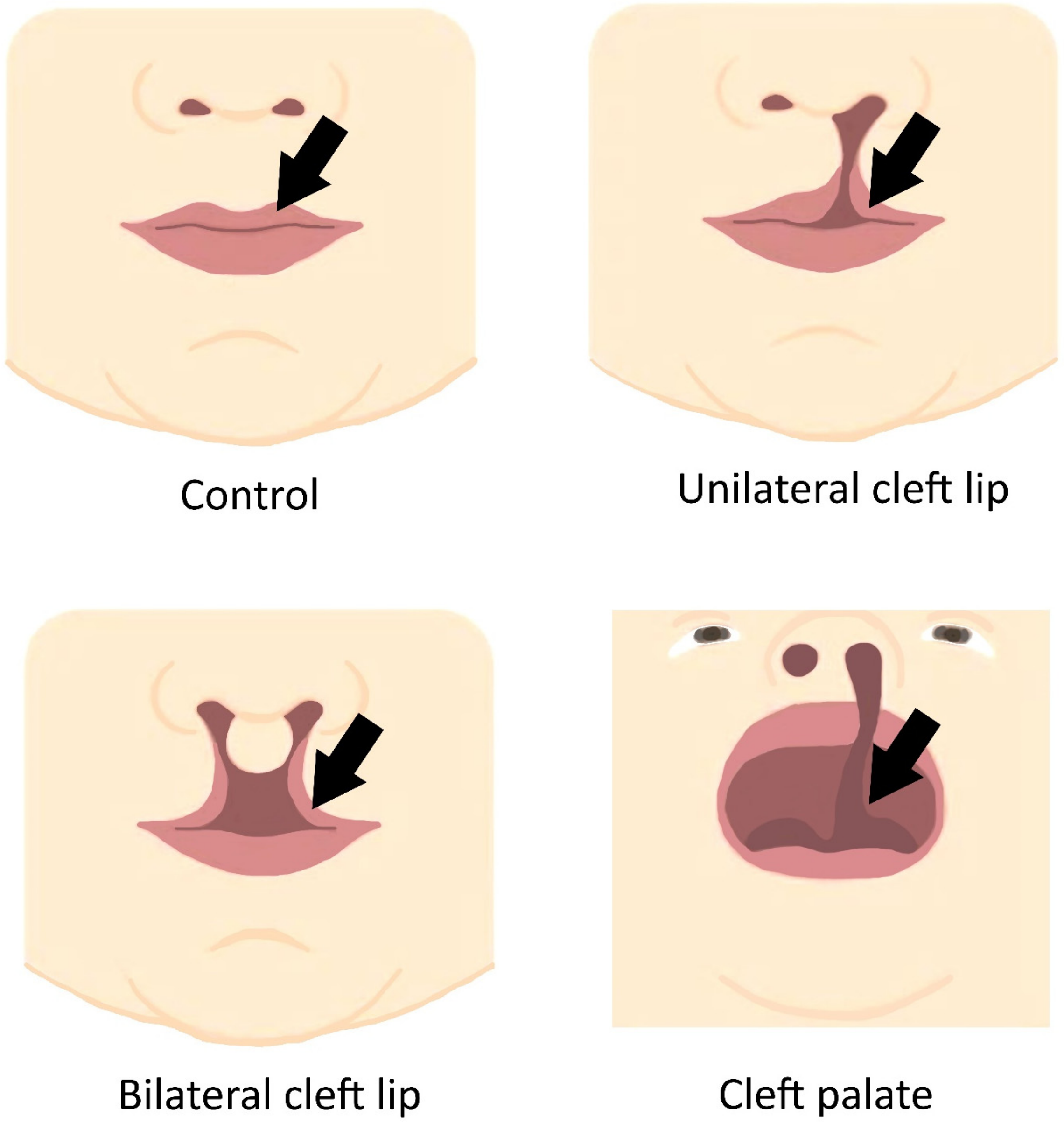

Figure S1. Localization of tissue used for the immunohistochemical evaluation of cleft affected tissue (arrows).

(Adapted from: https://www.shutterstock.com/image-vector/cleft-lip-birth-defect-baby-260nw-1964613478.jpg).
