# Supplementary material for: Characterization of SHH, SOX3, WNT3A and WNT9B Proteins in Human Non-Syndromic Cleft Lip and Palate Tissue
Source: Dent J (Basel). 2023 Jun 9;11(6):151. doi: 10.3390/dj11060151 (PMC10297181; doi:10.3390/dj11060151)
Supplement: Supplementary file 1 [file dentistry-11-00151-s001.zip › Supplementary material 2_Semiquantitative evaluation in 5 visual fields.pdf]

Supplementary material 2

Figure S2. Examples of semiquantitative evaluation of SHH, SOX3, WNT3A and WNT9B immunohistochemistry of cleft affected tissue and control tissue within 5 visual fields.

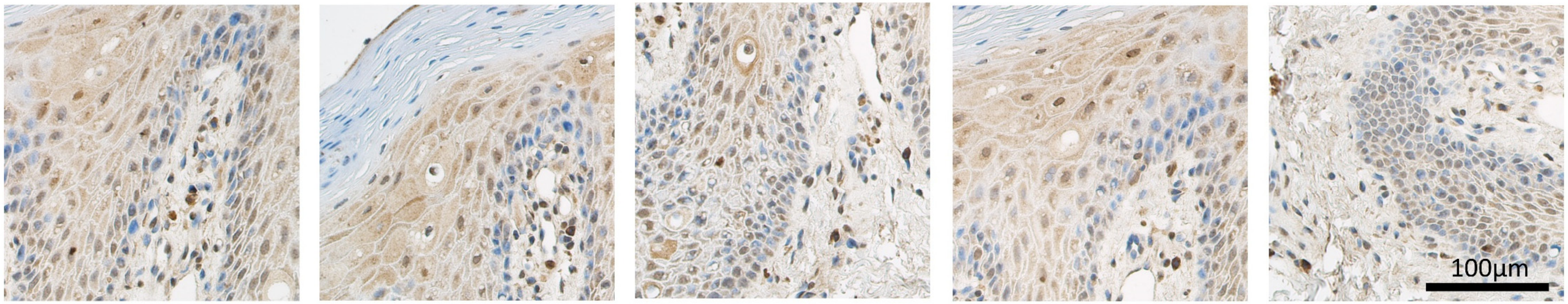

1A 1B 1C 1D 1E

Figure 1. Five visual fields of Sonic Hedgehog (SHH) immunohistochemistry from a single slide of unilateral cleft lip tissue.

(1A) Numerous (+++) SHH positive epithelial cells and a moderate (++) number of SHH immunoreactive cells in connective tissue, SHH immunohistochemistry, 400x.

(1B) A moderate (++) number of SHH positive epithelial cells and connective tissue cells, SHH immunohistochemistry, 400x.

(1C) Moderate to numerous (++/+++) SHH positive epithelial cells and a moderate (++) number of SHH positive cells in connective tissue, SHH immunohistochemistry, 400x.

(1D) Moderate to numerous (++/+++) SHH positive epithelial cells and a few to moderate (+/++) number of SHH positive cells in connective tissue, SHH immunohistochemistry, 400x.

(1E) Moderate to numerous (++/+++) SHH positive epithelial cells and a few to moderate (+/++) number of SHH positive connective tissue cells, SHH immunohistochemistry, 400x.

Table 1. Evaluation of SHH immunoreactivity from 5 visual fields within a single slide.

Visual field	Epithelium	Connective tissue
1A	+++	++
1B	++	++
1C	++/+++	++
1D	++/+++	+/++
1E	++/+++	+/++
**Median**	++/+++	++

Abbreviations: SHH – Sonic Hedgehog; +/++ - few to moderate number of immunoreactive cells in the visual field; ++ - moderate number of immunoreactive cells in the visual field; ++/+++ - moderate to numerous immunoreactive cells in the visual field; +++ - numerous immunoreactive cells in the visual field.

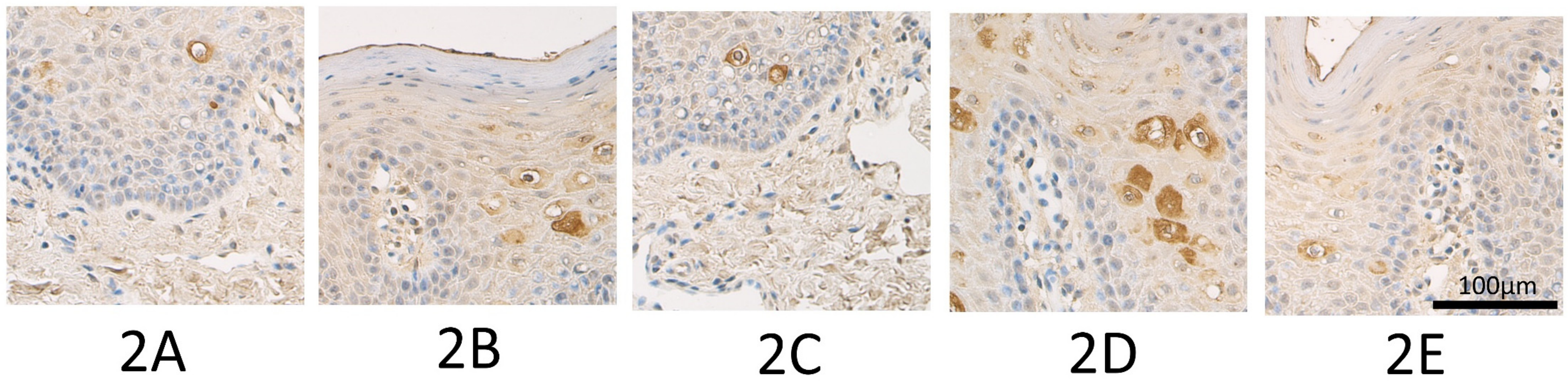

Figure 2. Five visual fields of SRY-box Transcription factor 3 (SOX3) immunohistochemistry from a single slide of bilateral cleft lip tissue.
(2A) Numerous (++/+++) SOX3 positive epithelial cells and a few (+) SOX3 positive connective tissue cells, SOX3 immunohistochemistry, 400x.
(2B) Numerous (+++) SOX3 positive epithelial cells and a few to moderate (+/++) SOX3 positive connective tissue cells, SOX3 immunohistochemistry, 400x.
(2C) Moderate to numerous (++/+++) SOX3 positive epithelial cells and a few (+) SOX3 positive cells in connective tissue, SOX3 immunohistochemistry, 400x.
(2D) Moderate to numerous (++/+++) SOX3 positive epithelial cells and a few (+) SOX3 positive cells in connective tissue, SOX3 immunohistochemistry, 400x.
(2E) Numerous (+++) SOX3 positive epithelial cells and a few to moderate (+/++) number of SOX3 positive connective tissue cells, SOX3 immunohistochemistry, 400x.

Table 2. Evaluation of SOX3 immunoreactivity from 5 visual fields within a single slide.

**Visual field**	**Epithelium**	**Connective tissue**
2A	+++	+
2B	+++	+/++
2C	++/+++	+
2D	++/+++	+
2E	+++	+/++
**Median**	+++	+

Abbreviations: SOX3 – SRY-box Transcription factor 3; + - a few immunoreactive cells in the visual field; +/++ - few to moderate number of immunoreactive cells in the visual field; ++ - moderate number of immunoreactive cells in the visual field; ++/+++ - moderate to numerous immunoreactive cells in the visual field; +++ - numerous immunoreactive cells in the visual field.

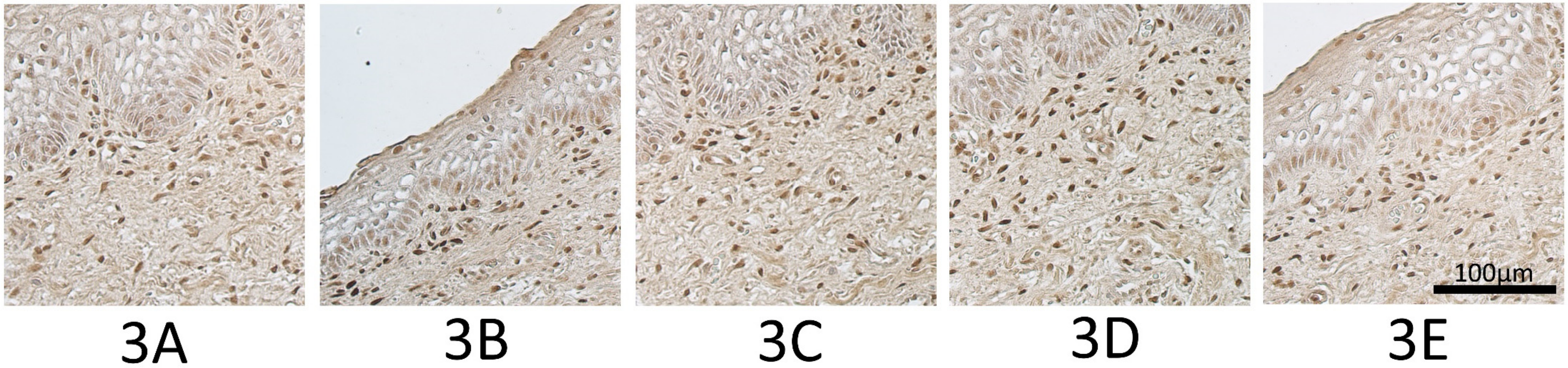

Figure 3. Five visual fields of WNT Family Member 3A (WNT3A) immunohistochemistry from a single slide of control tissue.

(3A) Moderate to numerous (++/+++) WNT3A positive epithelial cells and numerous (+++) WNT3A positive connective tissue cells, WNT3A immunohistochemistry, 400x.

(3B) Moderate to numerous (++/+++) WNT3A positive epithelial cells and numerous (+++) WNT3A positive connective tissue cells, WNT3A immunohistochemistry, 400x.

(3C) Moderate to numerous (++/+++) WNT3A positive epithelial cells and numerous (+++) WNT3A positive cells in connective tissue, WNT3A immunohistochemistry, 400x.

(3D) Moderate (++) number of WNT3A positive epithelial cells and numerous (+++) WNT3A positive cells in connective tissue, WNT3A immunohistochemistry, 400x.

(3E) Moderate to numerous (++) WNT3A positive epithelial cells and numerous (+++) WNT3A positive connective tissue cells, WNT3A immunohistochemistry, 400x.

Table 3. Evaluation of WNT3A immunoreactivity from 5 visual fields within a single slide.

Visual field	Epithelium	Connective tissue
3A	++/+++	+++
3B	++/+++	+++
3C	++/+++	+++
3D	++	+++
3E	++/+++	+++
**Median**	++/+++	+++

Abbreviations: WNT3A – WNT Family Member 3A; ++ - moderate number of immunoreactive cells in the visual field; ++/+++ - moderate to numerous immunoreactive cells in the visual field; +++ - numerous immunoreactive cells in the visual field

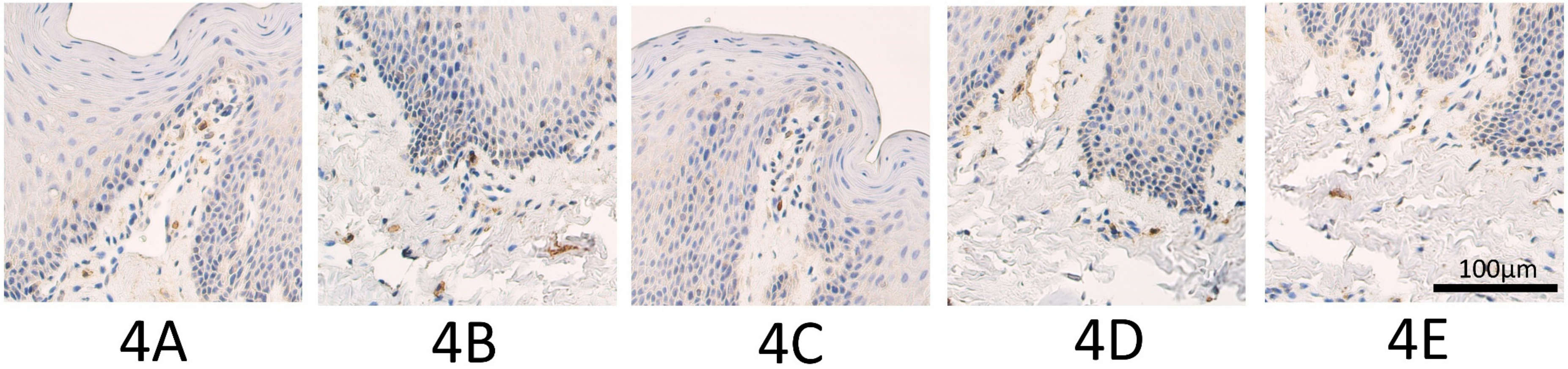

Figure 4. Five visual fields of WNT Family Member 9B (WNT9B) immunohistochemistry from a single slide of cleft palate tissue.

(3A) Moderate (++) number of weakly stained WNT9B positive epithelial cells and a few to moderate (+/++) number of WNT9B positive connective tissue cells, WNT9B immunohistochemistry, 400x.

(3B) Moderate to numerous (++/+++) weakly stained WNT9B positive epithelial cells and a few to moderate (+/++) WNT9B positive connective tissue cells, WNT9B immunohistochemistry, 400x.

(3C) Moderate (++) number of WNT9B positive epithelial cells and a few (+) WNT9B positive cells in connective tissue, WNT9B immunohistochemistry, 400x.

(3D) Moderate to numerous (++/+++) weakly stained WNT9B positive epithelial cells and a few (+) WNT9B positive cells in connective tissue, WNT9B immunohistochemistry, 400x.

(3E) Few to moderate (+/++) number of WNT9B positive epithelial cells and a few (+) WNT9B positive connective tissue cells, WNT9B immunohistochemistry, 400x.

Table 4. Evaluation of WNT9B immunoreactivity from 5 visual fields within a single slide.

**Visual field**	**Epithelium**	**Connective tissue**
4A	++	+/++
4B	++/+++	+/++
4C	++	+
4D	++/+++	+
4E	+/++	+
**Median**	++	+

Abbreviations: WNT9B – WNT Family Member 9B; + - a few immunoreactive cells in the visual field; +/++ - few to moderate number of immunoreactive cells in the visual field; ++ - moderate number of immunoreactive cells in the visual field; ++/+++ - moderate to numerous immunoreactive cells in the visual field.
